# Global Implications for COVID-19 Vaccine Series Completion: Insights from Real-World Data from the United States

**DOI:** 10.3390/vaccines10091561

**Published:** 2022-09-19

**Authors:** Jessica K. DeMartino, Ruibin Wang, Cindy Y. Chen, Nina Ahmad, Brahim Bookhart, Laurene Mascola

**Affiliations:** 1 Janssen Scientific Affairs, LLC, Titusville, NJ 08560, USA; 2 Janssen Medical Affairs, Titusville, NJ 08560, USA; 3 Health Officer Consultant, Vernon, CA 90058, USA

**Keywords:** COVID-19 vaccine, vaccine adherence, series completion, electronic health record data

## Abstract

This retrospective cohort analysis leveraged vaccination data for BNT162b2, mRNA-1273, and Ad26.COV2.S in the United States from the Komodo Healthcare Map database, the TriNetX Dataworks USA Network, and Cerner Real-World EHR (electronic health record) Data to evaluate rates of adherence to and completion of COVID-19 vaccination series (November 2020 through June 2021). Individuals were indexed on the date they received the first dose of a COVID-19 vaccine, with an adherence follow-up window of 42 days. Adherence/completion rates were calculated in the overall cohort of each database and by month of initiation and stratified by age, race/ethnicity, and urban/rural status. Overall adherence and completion to 2-dose COVID-19 mRNA vaccine schedules ranged from 79.4% to 87.4% and 81.0% to 89.2%, respectively. In TriNetX and Cerner, mRNA-1273 recipients were generally less adherent compared with BNT162b2 across sociodemographic groups. In Komodo, rates of adherence/completion between mRNA-1273 and BNT162b2 were similar. Adherence/completion were generally lower in younger (<65 years) versus older recipients (≥65 years), particularly for mRNA-1273. No other sociodemographic-based gaps in vaccine adherence/completion were identified. These data demonstrate high but incomplete adherence to/completion of multidose COVID-19 vaccines during initial vaccine rollout in the United States. Multidose schedules may contribute to challenges associated with successful global vaccination.

## 1. Introduction

During the ongoing severe acute respiratory syndrome coronavirus 2 (SARS-CoV-2) pandemic, over 550 million confirmed cases of coronavirus disease 2019 (COVID-19) have been reported worldwide, with >6.3 million reported COVID-19–related deaths, from emergence of the virus in 2019 through July 2022 [1]. In the United States, >88 million cases of COVID-19 have been reported, resulting in >4.9 million hospitalizations and >1 million deaths associated with COVID-19 [2,3].

In response to the pandemic, several vaccine candidates for the prevention of COVID-19 were rapidly developed and approved for use in the United States, including 2 messenger RNA (mRNA) vaccines (BNT162b2 [Comirnaty, Emergency Use Authorization (EUA) December 2020, approved August 2021], Pfizer-BioNTech, and mRNA-1273 [Spikevax, EUA December 2020, approved January 2022], Moderna) and 1 viral vector vaccine (Ad26.COV2.S [EUA February 2021], Janssen). Current guidance as of July 2022 from the Centers for Disease Control and Prevention (CDC) recommends administration of mRNA vaccines in a homologous 2-dose schedule with a booster dose administered ≥5 months after completing the primary series in those aged ≥5 years; adults aged ≥50 years can receive a second booster ≥4 months after the first booster. The recommended interval between dose 1 and dose 2 for BNT162b2 is 3 to 8 weeks, while the recommended interval between doses of mRNA-1273 is 4 to 8 weeks [4]. Ad26.COV2.S is administered as a single dose with a heterologous booster recommended ≥2 months after the primary dose [4,5,6]. 

In the United States, adult vaccination programs have historically experienced challenges, including difficulty adhering to multidose schedules [7,8,9,10]. As of July 2022, only 67.1% of the eligible US population had completed a primary vaccination series (i.e., 2 doses of an mRNA vaccine or 1 dose of Ad26.COV2.S) against COVID-19, with 77.9% having received at least 1 dose [11]. Booster vaccinations are necessary to protect against hospitalization and death that result from variant-driven waves of COVID-19; however, as of July 2022, 51.9% of those eligible for a first booster dose have not received one and only 28.5% of those eligible for a second booster dose have received one [11]. Globally, only 66% of the population has completed any COVID-19 primary vaccination series [12]. Only 16.2% of people in low-income countries have received 1 dose [12]. 

Given the challenges with adherence to multidose vaccine regimens among adults in the United States and the potential for reduced vaccine effectiveness due to non-adherence to multidose vaccine schedules, it is critical to investigate potential racial, ethnic, or other sociodemographic drivers [13]. In this study, we used real-world data gathered from 3 healthcare databases to assess adherence to and completion of primary vaccine schedules in adults aged ≥18 years living in the United States overall and within predefined sociodemographic subgroups by age, location, and race/ethnicity.

## 2. Materials and Methods

We leveraged data from the Komodo Healthcare Map, TriNetX Dataworks USA Network, and Cerner Real-World EHR (electronic health record) Data (Table S1). 

### 2.1. Komodo Healthcare Map

The Komodo Healthcare Map records de-identified claim-based healthcare encounters from 320 million insured individuals throughout the United States (2015–2022). Komodo is a nationally representative claims database that includes datasets from provider visits, laboratory tests, procedures, imaging, and prescriptions. The database is automatically refreshed monthly and is representative across age and geographic location of the insured US population. In this study, patients were identified in the Komodo database from 11 December 2020 to 21 June 2021.

### 2.2. TriNetX Dataworks USA

TriNetX records de-identified EHR-based clinical data from >137 million insured and uninsured patients. The data used in this study were collected on 4 August 2021 from the TriNetX Dataworks USA Network, which provided access to electronic medical records. Forty-four US-based healthcare organizations (HCOs) contributed data to the Dataworks USA Network database and allowed TriNetX to download their data for research purposes. The majority of HCOs that provide TriNetX with data are adult, acute care, academic medical institutions. Near real time–refreshed longitudinal data are available for demographics, diagnoses, medications, procedures, and lab measurements. Patients were identified in TriNetX from 11 December 2020 to 29 June 2021. 

### 2.3. Cerner Real-World EHR Data

Cerner Real-World EHR Data are extracted from the EHRs of >100 health systems across the United States. Date and time-stamped de-identified data from insured and uninsured patients are available for demographics, pharmacy encounters, clinical and microbiology laboratory tests, admissions, and billing information from affiliated inpatient, outpatient, and emergency room settings. Data is refreshed multiple times each month. Patients were identified in the Cerner database from 6 November 2020 to 20 May 2021.

### 2.4. Study Design

**Figure 1:**
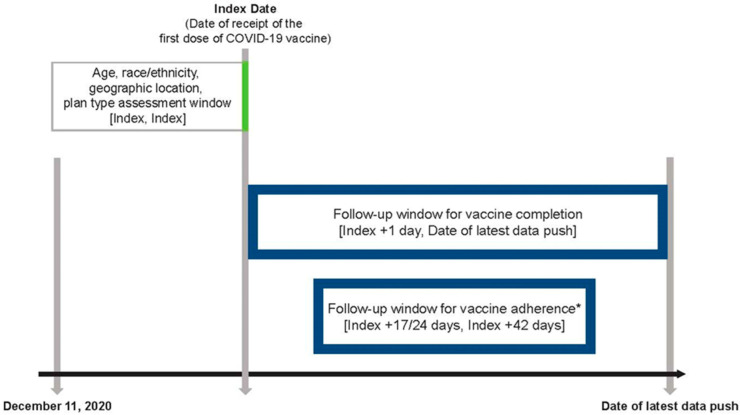
The study design. * Assessment period for adherence was determined based on FDA-approved dosing schedule and CDC guidance at the time of the study. COVID-19, coronavirus disease 2019; CDC, Centers for Disease Control and Prevention; FDA, US Food and Drug Administration.

Follow-up windows and adherence were based on the US Food and Drug Administration (FDA)–approved dosing schedule and CDC guidelines for 2-dose mRNA vaccines at the time of the study. Patient identification in Komodo and TriNetX began 11 December 2020, the date that the first COVID-19 vaccine (BNT162b2) received FDA emergency use authorization. The index date (Day 0) was defined as the calendar date that an individual received their first dose of a COVID-19 vaccine (Figure 1). Vaccine recipients were considered adherent to the 2-dose schedule if they received their second dose within the follow-up window, defined as the index date +17 to +42 days (BNT162b2) or the index date +24 to +42 days (mRNA-1273). Completion of the 2-dose vaccine schedule was defined as receipt of the second dose within the follow-up window of the index date +1 day to the date of latest data availability from each database. Those receiving 2 different brands of a COVID-19 vaccine were classified as “did not complete the 2-dose vaccine schedule.” For those receiving 2 homologous doses, the interval between doses was calculated and vaccine recipients were categorized into the following mutually exclusive groups according to the between-dose intervals: early, during the recommended interval, after the recommended interval but within the allowable interval, and late (Figure S1) [13].

### 2.5. Data Analysis

The proportion of vaccinated individuals in the overall analytical cohort and within subgroups by age (Komodo, TriNetX, and Cerner), race/ethnicity (TriNetX and Cerner), and urban/rural status (Komodo) was estimated. For BNT162b2 and mRNA-1273, the adherence and completion rates and corresponding 95% confidence intervals (CIs) were calculated in the overall analytical cohort and by month of initiation. Vaccine adherence and completion data were stratified by age (12–17 [BNT162b2 only], 18–22, 23–26, 27–39, 40–64, 65–74, 75–84, and ≥85 years), race (White, Black or African American, Asian, American Indian or Alaska Native, Other [Cerner only], and Unknown), ethnicity (Not Hispanic or Latino, Hispanic or Latino, and Unknown), and urban/rural status (large central metro, large fringe metro, medium metro, small metro, micropolitan, and non-core). Logistic regression analyses were performed to assess the association between vaccine adherence or completion and sociodemographic factors. Descriptive clustering analyses were performed to create national maps of county-level COVID-19 vaccine series adherence and completion. Statistical analyses were performed in Python Version 3.9 and SAS^®^ Studio 3.7 (Enterprise Edition). 

## 3. Results

### 3.1. Demographics of Vaccine Recipients 

In Komodo, 35,036,385 (male, 46.4%; female, 53.6%) vaccine recipients were identified in the study window; among these, 48.5% received BNT162b2, 49.0% received mRNA-1273, 2.1% received Ad26.COV2.S, and 0.4% received multiple brands of vaccines. A total of 1,319,537 patients in the TriNetX database had received ≥1 dose of a COVID-19 vaccine (male, 42.6%; female, 56.1%) from 35 HCOs. Of these, 82.0% received BNT162b2, 16.2% received mRNA-1273, 1.8% received Ad26.COV2.S, and <0.1% received multiple brands of vaccines. In the Cerner database, 2,158,526 individuals had received ≥1 dose of a COVID-19 vaccine (male, 41.7%; female, 57.8%); among these, 61.0% received BNT162b2, 36.0% received mRNA-1273, and 3.0% received Ad26.COV2.S (Table S2). 

### 3.2. Overall Vaccine Adherence and Completion

Adherence to the 2-dose schedule for mRNA vaccines in Komodo was 79.4% overall, 79.5% among those receiving BNT162b2, and 79.4% among those receiving mRNA-1273 (Table 1). Completion was 81.0% overall; 80.6% and 81.2% of those receiving BNT162b2 and mRNA-1273, respectively, completed the series. Among TriNetX vaccine recipients, overall adherence was 85.6% (BNT162b2, 88.1%; mRNA-1273, 72.9%). Overall completion was 86.5%; 88.7% of BNT162b2 recipients and 75.3% of mRNA-1273 recipients completed their COVID-19 vaccination schedules. In the Cerner database, adherence was 87.4% overall, 90.5% among those receiving BNT162b2, and 82.1% among those receiving mRNA-1273; 89.2% (overall), 91.2% (BNT162b2), and 86.0% (mRNA-1273) of recipients completed the series. The majority of vaccinees (>85%) received a second dose during the recommended window (Table S3).

### 3.3. Vaccine Adherence and Completion by Age

**Figure 2:**
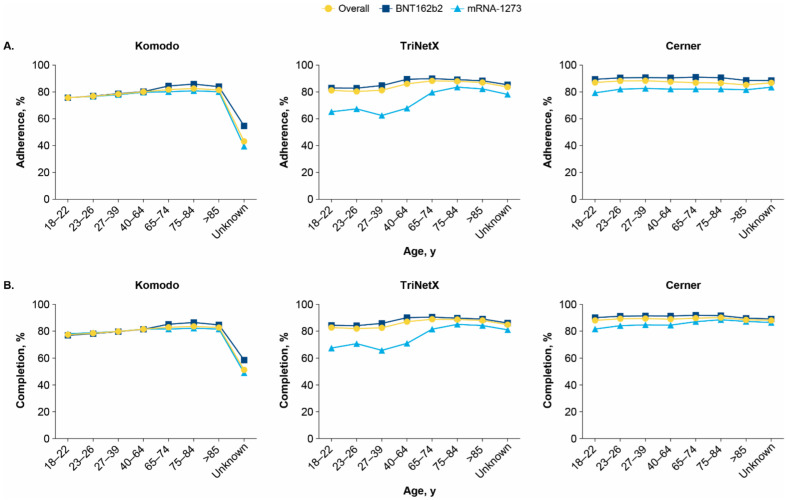
(**A**) Adherence and (**B**) completion stratified by age (years) in the Komodo, TriNetX, and Cerner databases.

In Komodo, adherence and completion varied by age group, with younger recipients (<65 years) exhibiting lower rates compared with older recipients (≥65 years); the lowest average adherence was observed among recipients aged 18 to 22 years (75.7%) and highest adherence was observed in those aged 75 to 84 years (82.6%). In the TriNetX database, adherence was lower in younger age groups, particularly for mRNA-1273. Compared with those receiving BNT162b2, adherence and completion were lower for those receiving mRNA-1273 in all age groups of the Cerner database; younger recipients had lower adherence and completion than older recipients (Figure 2 and Table S4).

### 3.4. Vaccine Adherence and Completion by Race, Ethnicity, Urban/Rural Status, and Geographic Region

**Figure 3:**
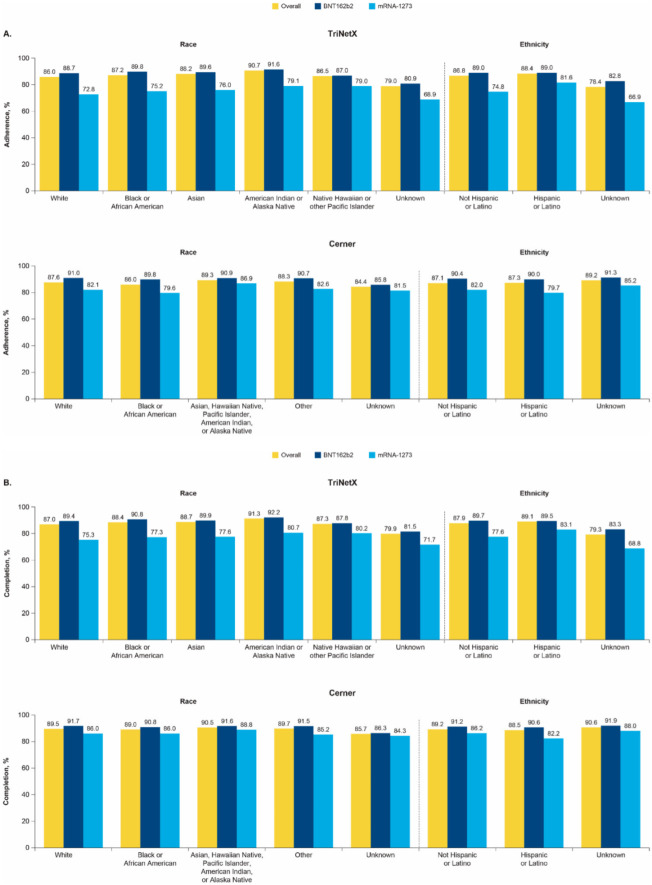
(**A**) Adherence and (**B**) completion stratified by race and ethnicity in the TriNetX and Cerner databases.

Differences in vaccine adherence or completion between race/ethnicity subgroups were small (Figure 3). COVID-19 vaccination adherence and completion did not vary substantially by urban/rural status (Figure S2). Most US counties with available data had series adherence and completion rates between 80% to 90%, which were higher than the COVID-19 vaccine coverage rates (i.e., the percentage of the US population who received at least 1 dose) reported by the CDC (Figure S3) [14]. 

### 3.5. Vaccine Adherence and Completion by Index Month

**Figure 4:**
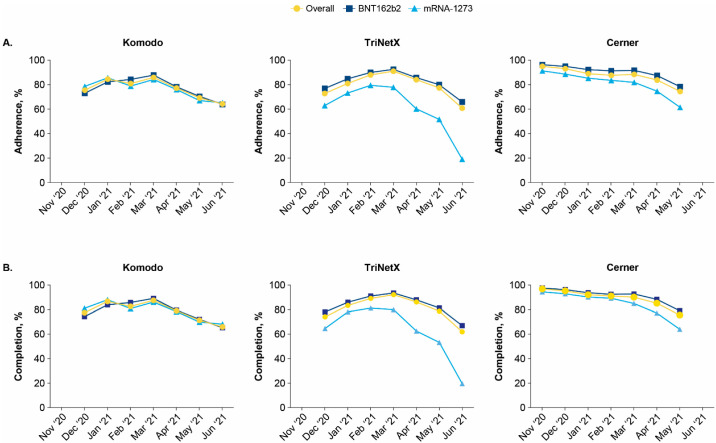
(**A**) Adherence and (**B**) completion by index month in the Komodo, TriNetX, and Cerner databases. Individuals included in Komodo and TriNetX databases were identified from December 2020 to June 2021. Individuals included in the Cerner database were identified from November 2020 to May 2021.

When evaluating by index month, ranging from December 2020 to June 2021, overall adherence to vaccination schedules in Komodo was 76% among those initiating vaccination in December 2020, peaked at 86% for those with an index month of March 2021, and declined to 64% for those with an index month of June 2021. Decreases in adherence were similar among recipients of BNT162b2 (73% to 64%, for December and June index months, respectively) and mRNA-1273 (79% to 65%, respectively). Among those initiating vaccination in December 2020, 77% completed the series compared with 66% of those initiating in June 2021; completion by index month for BNT162b2 and mRNA-1273 recipients decreased from 74% to 65% and 81% to 68%, respectively (Figure 4 and Table S5). Similar trends of decreasing adherence and completion were observed across age groups when evaluating by index month. Completion rates peaked for patients aged ≤65 years indexed in March 2021, while completion rates peaked for those aged ≥65 years indexed in January 2021 (Figure S4).

When the TriNetX database was evaluated by index month, COVID-19 vaccination adherence and completion were lower for mRNA-1273 compared with BNT162b2 and decreased substantially from April 2021 through June 2021. The lower vaccine adherence and completion observed among younger recipients (<65 years) compared with older recipients (≥65 years) varied by index month. Among those in the Cerner database, overall adherence and completion declined between November 2020 and May 2021; recipients of mRNA-1273 had a greater decrease in adherence/completion compared with BNT162b2 (Figure 4 and Table S5). Similar decreases in adherence and completion by index month were observed in subgroups stratified by age and race (Figures S4 and S5).

## 4. Discussion

In this large, retrospective cohort analysis using data from 3 healthcare claims and EHR databases, we identified consistent trends in COVID-19 vaccination series adherence and completion in the United States, with rates reaching 80% to 90%. In the TriNetX and Cerner databases, mRNA-1273 recipients were generally less adherent and completed their vaccination schedules at a lower rate compared with recipients of BNT162b2 across sociodemographic groups. Lower mRNA-1273 completion rates in the TriNetX database may have occurred because the majority of data in TriNetX comes from academic medical centers with systematic data delivery; these academic institutions may have been more likely to administer BNT162b2 because they have the necessary cold chain storage capacity. In the Komodo database, adherence and completion were similar between the 2 vaccines, which agrees with CDC data indicating that, between December 2020 and February 2021, similar proportions of BNT162b2 (87.6%) and mRNA-1273 (88.7%) vaccine recipients completed their COVID-19 vaccination series [13].

When stratifying by sociodemographic factors, COVID-19 vaccine adherence/completion were observed to be lower in younger vaccine recipients (<65 years) compared with older recipients (≥65 years). These findings are consistent with a large study of Veterans Affairs data, in which older age was identified as an independent predictor of vaccine completion, and the higher overall rates of completion (95%) observed in that study may be partly explained by the older population [15]. Older adults may have been more adherent to 2-dose COVID-19 vaccine schedules because they possess adequate knowledge of COVID-19 compared with younger populations [16,17], and thus have greater adherence to preventive health measures recommended by public health agencies [17,18].

Our findings agree with the CDC data that demonstrated minimal differences in COVID-19 vaccination completion between racial groups [11]. These findings may be promising indicators of series completion; however, we cannot make any conclusions regarding vaccine uptake/coverage stratified by race because our study accounted only for those who initiated a vaccine series.

Although differences by urban/rural status have been observed in vaccine coverage as well as in series completion [15,19,20], our study did not identify differences in completion based on urban/rural status. We were not able to observe any differences by urban/rural status that may have become more pronounced in later months because our study window was contained within the early months of vaccination rollout. 

Vaccine adherence decreased substantially for recipients with later index dates, particularly among those receiving mRNA-1273 in the TriNetX and Cerner databases, which may have impacted the overall trends. Many early vaccine recipients were healthcare workers or older adults (≥65 years) who had higher rates of vaccination series adherence and completion; vaccine availability date also varied by state for the general population, which may have contributed to the differences in adherence by index date. Differences found by index date may also reflect the waves of COVID-19 seen throughout 2021, which potentially impacted vaccine uptake. The trend of reduced adherence and completion with later index dates in this study aligns with the overall trend in the United States, where a decrease was observed in vaccine administration after the initial rollout [12,21].

The study period for this analysis focused on the pre-Delta stage of the pandemic, which had high morbidity and mortality rates. In the United States, extensive governmental resources and broad access to healthcare were relied on to effectively roll out mass COVID-19 vaccination programs during the pandemic. Despite the ease of access and ample resources available to the US population relative to other countries, adherence and completion did not reach 100% for multidose vaccines. Lack of adherence/completion of a COVID-19 vaccine regimen leaves some people vulnerable to severe illness, hospitalization, and death. Given the vaccination challenges resource-rich countries have faced in a pandemic setting [22], well-informed strategies to improve vaccine adherence both within the United States and globally are critical.

Although this study evaluated US data, the results have important implications for non-US populations. As of 2022, there remain many challenges to successful vaccination of the global population. Many low- and middle-income countries (LMICs) face significant challenges in acquiring and distributing COVID-19 vaccines. For example, when premarket purchase commitments were announced in November 2020, just over half (51%) of all premarket-purchased COVID-19 vaccine doses were purchased by high-income countries, representing only 14% of the global population [23]. LMICs generally rely on the COVID-19 Vaccines Global Access (COVAX) Facility, which is financed by higher-income country donors, to obtain vaccines [24]. Additionally, many LMICs do not have established routine immunization programs for adults to leverage for vaccine distribution [25]. In the United States, as of July 2022, the CDC recommends that non-immunocompromised persons aged ≥5 years receive a single booster, and a second booster is available for those aged ≥50 years in order to enhance protection against emerging variants of concern; this strategy may present a challenge globally [26]. As of July 2022, only 28.5% of those eligible in the United States have received a second booster, indicating low uptake of fourth doses of mRNA vaccines [11]. Low booster coverage is particularly concerning as Omicron BA.4/5 subvariants have emerged. Although lower vaccine efficacy against Omicron has been observed compared with other variants, such as Delta, initial data suggest that boosters will provide protection against severe disease, highlighting the need for increased booster uptake [27,28,29,30]. 

Because completion rates for vaccines with 2-dose primary regimens were not 100% and single doses of these intended multidose regimens may be less effective, adherence and uptake may be improved by implementation of an effective vaccination regimen with the fewest doses [9,10,31,32]. Previous studies of other vaccine programs have shown that the number of doses required in multidose vaccine schedules may influence vaccination adherence and rates of series completion. For example, use of a 2-dose hepatitis B vaccination series resulted in better adherence relative to a 3-dose schedule (completion rates at 1 year, 60.5% vs. 32.3%, respectively) [10]. Similarly, despite recommendations for adults aged ≥65 years to be vaccinated against pneumococcal disease, a retrospective study demonstrated low adherence to 2-dose pneumococcal vaccine series completion (16.6%), as well as low series initiation (34.3%) [33]. A primary barrier to vaccination against human papilloma virus (HPV) among adults was cited as the inconvenience of returning for additional doses [34]. Recent survey data suggests that series completion of the 3-dose HPV vaccine among college-aged adults was 17.4%, compared with 47% who initiated the series [35]. Completion rates of HPV vaccine series among adolescents in the United States have increased following a change to the recommended vaccination schedule from 3 doses to 2 doses in those initiating the series under the age of 15 [36]. Enabling flexible-dosing schedules can improve adherence for 2-dose vaccination series [8], and fewer required doses in the recommended vaccine schedule may also improve cost-effectiveness [37,38,39].

Effective and durable COVID-19 vaccines with fewer doses may play a critical role in global vaccination efforts. The FDA has limited the authorized use of the single-dose vaccine Ad26.COV2.S to individuals ≥18 years for whom other FDA-authorized or approved COVID-19 vaccines are not accessible or clinically appropriate [5]. This aligns with the Advisory Committee on Immunization Practices recommendation of preferential use of multidose mRNA COVID-19 vaccines versus single-dose (primary regimen) Ad26.COV2.S in light of a more favorable risk-benefit balance and the wide availability of mRNA vaccines in the United States as of June 2022. They concluded that the benefits of Ad26.COV2.S in some situations (e.g., those contraindicated to mRNA vaccines or those without access to mRNA vaccines) may outweigh the risk of potential rare serious adverse events (specifically, Guillain-Barré syndrome and thrombosis with thrombocytopenia) [40].

The major strength of this study was the use of 3 data sources, which had a high level of completeness for race and ethnicity data and near real time–data refresh, allowing for in-depth examination of adherence and completion of COVID-19 vaccination schedules. Limitations of this study included possible underrepresentation of institutionalized and uninsured individuals and those who may opt out of COVID-19 vaccination. Because claims and EHR data are not collected for research purposes, our results may also underestimate vaccine series completion for those who received 2 doses of an mRNA COVID-19 vaccine with only 1 encounter recorded in the database. Due to the unavailability of predictors for not receiving healthcare services (e.g., financial challenge, loss of enrollment in healthcare plans, etc.), we were not able to correct for this bias using censoring weights. Additionally, HCOs contributing to TriNetX were skewed towards academic, acute care settings. Reasons for non-adherence and incompletion in this study are unknown. It is possible that challenges in the beginning of COVID-19 vaccine roll-out and scheduling may have contributed to underestimation of vaccine series completion. Given that the vaccines were free-of-cost, it is possible some people may have given no insurance data.

Overall, our results show that there were some challenges regarding multidose COVID-19 vaccination adherence and completion in the United States, despite extensive governmental and healthcare resources, with implications for global vaccination efforts. Flexible vaccination schedules or the availability of vaccines that achieve durable responses with fewer doses may facilitate COVID-19 vaccine adherence and completion and alleviate implementation constraints faced by some LMICs. Ultimately, different strategies for roll out and uptake of vaccines in a pandemic versus endemic setting will likely be necessary, as resource allocation may decline. This study, conducted in a large, representative population, may help inform future efforts to understand the lower rates of vaccination in younger age groups, whereby strategies might be developed to improve adherence in general. Although the role of social determinants of health (e.g., income, education, and health insurance) and social capital (e.g., community and institutional health) have been shown to contribute to vaccination disparities in a US dataset [41], their impact in the United States and globally represents an important target of further study. For future pandemics, strategies to improve vaccine adherence and completion will be critical to maximize mitigation of social, economic, and health-related impacts, both in the United States and globally.

## Figures and Tables

**Table 1:** Adherence and completion in the Komodo, TriNetX, and Cerner databases.

	Overall	BNT162b2	mRNA-1273
Adherence, % ^a^			
Komodo	79.4	79.5	79.4
TriNetX	85.6	88.1	72.9
Cerner	87.4	90.5	82.1
Completion, % ^a^			
Komodo	81.0	80.6	81.2
TriNetX	86.5	88.7	75.3
Cerner	89.2	91.2	86.0

^a^ Adherence and completion of Ad26.COV2.S primary vaccination was considered 100%. Komodo: *N* = 35,036,385 (BNT162b2, *n* = 17,001,191; mRNA-1273, *n* = 17,160,400; Ad26.COV2.S, *n* = 748,232; multiple vaccines, *n* = 126,562). TriNetX: *N* = 1,319,537 (BNT162b2, *n* = 1,082,365; mRNA-1273, *n* = 213,438; Ad26.COV2.S, *n* = 23,127; multiple vaccines, *n* = 607). Cerner: *N* = 2,158,526 (BNT162b2, *n* = 1,316,052; mRNA-1273, *n* = 777,015; Ad26.COV2.S, *n* = 64,259).

## Data Availability

The de-identified row-level data may be obtained through data licenses for Komodo Healthcare Map, TriNetX Dataworks USA Network, and Cerner Real-World EHR Data and are not publicly available.

## Supplementary Materials

The following supporting information can be downloaded at: https://www.mdpi.com/article/10.3390/vaccines10091561/s1, Table S1: Objective-specific database inclusion criteria; Table S2: Demographics of vaccine recipients; Table S3: Distribution of between-dose intervals for the Komodo, TriNetX, and Cerner databases; Table S4: Rates of adherence and completion stratified by age in the Komodo, TriNetX, and Cerner databases; Table S5: Rates of adherence and completion stratified by index month in the Komodo, TriNetX, and Cerner databases; Figure S1: Recommended timeline for receipt of 2 doses of mRNA COVID-19 vaccines; Figure S2: Adherence and completion stratified by urban/rural status (Komodo); Figure S3: Adherence and completion by county in the Komodo database and nationwide COVID-19 vaccination coverage by county showing the percent of the total United States population with at least one dose; Figure S4: Adherence and completion by index month, stratified by age; Figure S5: Adherence and completion by index month, stratified by race.
