# Supplementary material for: Global Implications for COVID-19 Vaccine Series Completion: Insights from Real-World Data from the United States
Source: Vaccines (Basel). 2022 Sep 19;10(9):1561. doi: 10.3390/vaccines10091561 (PMC9502798; doi:10.3390/vaccines10091561)

Supplementary Materials

Representativeness of the Komodo Healthcare Map

Table S1. Objective-specific database inclusion criteria

Table S2. Demographics of vaccine recipients

Table S3. Distribution of between-dose intervals for the Komodo, TriNetX, and Cerner databases

Table S4. Rates of adherence and completion stratified by age (years) in the Komodo, TriNetX, and Cerner databases

Table S5. Rates of adherence and completion stratified by index month in the Komodo, TriNetX, and Cerner databases.

Figure S1. Recommended timeline for receipt of 2 doses of mRNA COVID-19 vaccines

Figure S2. Adherence and completion stratified by urban/rural status (Komodo)

Figure S3. Adherence and completion by county in the Komodo database and nationwide COVID-19 vaccination coverage by county showing the percent of the total United States population with at least one dose

Figure S4. Adherence and completion by index month, stratified by age

Figure S5. Adherence and completion by index month, stratified by race

Representativeness of the Komodo Healthcare Map

The representativeness of the Komodo database has been demonstrated in an internal comparative study conducted by Komodo Health. The age, sex, and geographic distributions of patients in Komodo's 2019 patient population were compared to those from the 2019 National Health Interview Survey (NHIS), an annual national survey conducted by the Centers for Disease Control and Prevention to monitor the health of the civilian noninstitutionalized population of the United States. The demographics of Komodo patients are highly comparable to those from the contemporary NHIS population as demonstrated by the close alignment in the statistics:

	Komodo	NHIS
**Breakdown by age groups, %**		
Age, y		
0-9	11.7	11.3
10-19	12.0	12.3
20-29	10.9	9.6
30-39	11.6	12.6
40-49	11.5	11.3
50-59	13.7	12.8
60-69	13.8	14.4
70-79	9.4	9.9
≥80	5.5	5.6
**Proportion of female patients, %**	55	54
**Breakdown by geographic locations, %**		
Northeast	19.5	16.9
South	38.0	36.5
Midwest	21.6	22.2
West	20.9	24.4

Supplementary Tables

Table S1: Objective-specific database inclusion criteria

Description	Database	Inclusion criteria
Brand distribution for the first dose of COVID-19 vaccine	Komodo<br>TriNetX<br>Cerner	All individuals who had at least 1 documented COVID-19 vaccine CPT/NDC/RxNorm code between Dec 11, 2020, and database-specific data cut date[a]
Completion and adherence to 2-dose COVID-19 vaccine	Komodo<br>TriNetX<br>Cerner<br>CDC[b]	All individuals who had at least 1 CPT/NDC/RxNorm code for COVID-19 vaccines with 2-dose schedule between Dec 11, 2020, and (Database-specific data cut date - 42d)[c]
Age gaps in vaccine completion and adherence	Komodo<br>TriNetX<br>Cerner<br>CDC[b]	
Geographic gaps in vaccine completion and adherence	Komodo	
Racial gap in vaccine completion and adherence	TriNetX<br>Cerner<br>CDC[b]	
Vaccine completion and adherence by payer type	Komodo	

[a]Data cut-off dates were as follows: Komodo Healthcare Map, August 2, 2021; TriNetX Dataworks USA Network, August 10, 2021; Cerner Real-World EHR Data, June 30, 2021.
[b]No inclusion criteria were applied to the CDC data, as it was downloaded and used as-is.
[c]Data cut-off dates were as follows: Komodo, June 21, 2021; TriNetX, June 29, 2021; Cerner, May 20, 2021.
CDC, Centers for Disease Control; CPT, Current Procedural Terminology; NDC, National Drug Code

Table S2: Demographics of vaccine recipients

	Komodo					TriNetX					Cerner			
**Demographic**	**Overall**	**BNT162b2**	**mRNA-1273**	**Ad26.COV2.S**	**Multiple Vaccines**	**Overall**	**BNT162b2**	**mRNA-1273**	**Ad26.COV2.S**	**Multiple Vaccines**	**Overall**	**BNT162b2**	**mRNA-1273**	**Ad26.COV2.S**
N	35,036,385	17,001,191	17,160,400	748,232	126,562	1,319,537	1,082,365	213,438	23,127	607	2,158,526	1,316,052	777,015	64,259
(% of overall)	-	(48.5)	(49.0)	(2.1)	(0.4)	-	(82.0)	(16.2)	(1.8)	(≤0.1)	-	(61.0)	(36.0)	(3.0)
Age, mean	49.3	44.8	53.6	49.6	49.7	54.1	53.6	57.4	48.2	53.4	-	-	-	-
(SD)	(19.3)	(19.7)	(18.1)	(16.4)	(15.9)	(19.3)	(19.5)	(18.3)	(16.4)	(18.4)				
Age group, n (%)														
18–22	2,121,647 (6.1)	1,283,614 (7.6)	787,020 (4.6)	44,918 (6.0)	6,095 (4.8)	50,480 (3.8)	44,096 (4.1)	5,006 (2.3)	1,353 (5.9)	25 (4.1)	87,931 (4.1)	64,668 (4.9)	20,291 (2.6)	2,947 (4.6)
23–26	1,639,178 (4.7)	842,359 (5.0)	755,220 (4.4)	36,349 (4.9)	5,250 (4.1)	51,345 (3.9)	41,919 (3.9)	8,223 (3.9)	1,186 (5.1)	17 (2.8)	77,200 (3.6)	54,809 (4.2)	20,111 (2.6)	2,261 (3.5)
27–39	6,129,155 (17.5)	3,128,979 (18.4)	2,841,856 (16.6)	135,731 (18.1)	22,589 (17.8)	200,885 (15.2)	164,263 (15.2)	31,490 (14.8)	5,026 (21.7)	106 (17.5)	294,423 (13.6)	204,061 (15.5)	81,050 (10.4)	9,201 (14.3)
40–64	14,975,055 (42.7)	7,059,354 (41.5)	7,444,314 (43.4)	401,260 (53.6)	70,127 (55.4)	486,938 (36.9)	399,341 (36.9)	75,604 (35.4)	11,729 (50.7)	264 (43.5)	811,085 (37.6)	505,714 (38.4)	272,669 (35.1)	32,289 (50.3)
65–74	5,018,098 (14.3)	1,776,030 (10.4)	3,138,681 (18.3)	88,320 (11.8)	15,067 (11.9)	275,846 (20.9)	226,277 (20.9)	46,957 (22.0)	2,520 (10.9)	92 (15.2)	468,252 (21.7)	245,318 (18.6)	211,323 (27.2)	11,290 (17.6)
75–84	2,509,277 (7.2)	837,560 (4.9)	1,636,807 (9.5)	29,777 (4.0)	5,133 (4.1)	163,915 (12.4)	128,157 (11.8)	34,761 (16.3)	942 (4.1)	55 (9.1)	275,201 (12.8)	142,069 (10.8)	128,368 (16.5)	4,539 (7.1)
≥85	854,602 (2.4)	288,855 (1.7)	553,137 (3.2)	11,407 (1.5)	1,203 (1.0)	36,238 (2.7)	28,341 (2.6)	7,625 (3.6)	248 (1.1)	24 (4.0)	87,660 (4.1)	44,862 (3.4)	41,244 (5.3)	1,481 (2.3)
Unknown	5,996 (≤0.1)	1,063 (≤0.1)	3,365 (≤0.1)	470 (0.1)	1,098 (0.9)	15,902 (1.2)	11,983 (1.1)	3,772 (1.8)	123 (0.5)	24 (4.0)	3,363 (0.2)	1,949 (0.2)	1,298 (0.2)	113 (0.2)
Sex, n (%)														
Male	16,258,100 (46.4)	7,907,495 (46.5)	7,916,043 (46.1)	374,335 (50.0)	60,227 (47.6)	562,618 (42.6)	469,581 (43.4)	81,188 (38.0)	11,575 (50.0)	274 (45.1)	900,983 (41.7)	551,458 (41.9)	320,122 (41.2)	28,972 (45.1)
Female	18,776,894 (53.6)	9,093,198 (53.5)	9,243,471 (53.9)	373,893 (50.0)	66,332 (52.4)	740,232 (56.1)	605,162 (55.9)	123,812 (58.0)	10,950 (47.3)	308 (50.7)	1,248,278 57.8)	758,774 (57.7)	453,741 (58.4)	34,997 (54.5)
Unknown	1,391 (≤0.1)	498 (≤0.1)	886 (≤0.1)	4 (≤0.1)	3 (≤0.1)	16,687 (1.3)	7,622 (0.7)	8,438 (4.0)	602 (2.6)	25 (4.1)	9,265 (0.4)	5,820 (0.4)	3,152 (0.4)	290 (0.5)
Race, n (%)[a]														

	Komodo					TriNetX					Cerner			
**Demographic**	**Overall**	**BNT162b2**	**mRNA-1273**	**Ad26.COV2.S**	**Multiple Vaccines**	**Overall**	**BNT162b2**	**mRNA-1273**	**Ad26.COV2.S**	**Multiple Vaccines**	**Overall**	**BNT162b2**	**mRNA-1273**	**Ad26.COV2.S**
White	-	-	-	-	-	875,826 (66.4)	714,320 (66.0)	146,368 (68.6)	14,743 (63.7)	395 (65.1)	1,563,531 (72.4)	934,709 (71.0)	584,984 (75.3)	42,905 (66.8)
Black or African American	-	-	-	-	-	192,815 (14.6)	156,504 (14.5)	33,426 (15.7)	2,774 (12.0)	111 (18.3)	146,343 (6.8)	88,351 (6.7)	52,260 (6.7)	5,677 (8.8)
Asian[b]	-	-	-	-	-	87,392 (6.6)	77,317 (7.1)	8,657 (4.1)	1,394 (6.0)	24 (4.0)				
American Indian or Alaska Native[d]	-	-	-	-	-	9,565 (0.7)	8,474 (0.8)	642 (0.3)	447 (1.9)	2 (0.3)	91,898 (4.3)	55,267 (4.2)	33,695 (4.3)	2,881 (4.5)
Native Hawaiian or other Pacific Islander[b]	-	-	-	-	-	2,789 (0.2)	2,470 (0.2)	167 (0.1)	151 (0.7)	1 (0.2)				
Other	-	-	-	-	-	-	-	-	-	-	192,482 (8.9)	130,324 (9.9)	54,429 (7.0)	7,635 (11.9)
Unknown	-	-	-	-	-	151,150 (11.5)	123,280 (11.4)	24,178 (11.3)	3,618 (15.6)	74 (12.2)	164,272 (7.6)	107,401 (8.2)	51,647 (6.7)	5,161 (8.0)
Ethnicity, n (%)[a]	-	-	-	-	-									
Not Hispanic or Latino	-	-	-	-	-	945,993 (71.7)	787,253 (72.7)	141,290 (66.2)	17,146 (74.1)	304 (50.1)	1,557,040 (72.1)	905,337 (68.8)	604,474 (77.8)	46,254 (72.0)
Hispanic or Latino	-	-	-	-	-	146,944 (11.1)	133,992 (12.4)	10,603 (5.0)	2,327 (10.1)	22 (3.6)	329,321 (15.3)	236,330 (18.0)	82,326 (10.6)	10,537 (16.4)
Unknown	-	-	-	-	-	226,600 (17.2)	161,120 (14.9)	61,545 (28.8)	3,654 (15.8)	281 (46.3)	272,165 (12.6)	174,385 (13.3)	90,215 (11.6)	7,469 (11.6)
Urban/rural status, n (%)[c]														
Large central metro	9,987,328 (28.5)	5,167,106 (30.4)	4,521,939 (26.4)	264,736 (35.4)	33,547 (26.5)	-	-	-	-	-	-	-	-	-

	Komodo					TriNetX					Cerner			
Demographic	Overall	BNT162b2	mRNA-1273	Ad26.COV2.S	Multiple Vaccines	Overall	BNT162b2	mRNA-1273	Ad26.COV2.S	Multiple Vaccines	Overall	BNT162b2	mRNA-1273	Ad26.COV2.S
Large fringe metro	7,706,888 (22.0)	3,698,288 (21.8)	3,837,102 (22.4)	130,304 (17.4)	41,194 (32.5)	-	-	-	-	-	-	-	-	-
Medium metro	6,737,311 (19.2)	3,139,339 (18.5)	3,463,896 (20.2)	112,283 (15.0)	21,793 (17.2)	-	-	-	-	-	-	-	-	-
Small metro	2,811,488 (8.0)	1,266,257 (7.4)	1,478,496 (8.6)	58,224 (7.8)	8,511 (6.7)	-	-	-	-	-	-	-	-	-
Micropolitan	2,318,863 (6.6)	781,047 (4.6)	1,473,611 (8.6)	56,174 (7.5)	8,031 (6.3)	-	-	-	-	-	-	-	-	-
Non-core	1,405,171 (4.0)	370,716 (2.2)	985,905 (5.7)	43,975 (5.9)	4,575 (3.6)	-	-	-	-	-	-	-	-	-
Unknown	4,069,336 (11.6)	2,578,438 (15.2)	1,399,451 (8.2)	82,536 (11.0)	8,911 (7.0)	-	-	-	-	-	-	-	-	-

[a]Data for race/ethnicity was only available for the TriNetX and Cerner databases.

[b]In the Cerner database, Asian, Hawaiian Native, Pacific Islander, American Indian, and Alaskan Native were grouped together.

[c]Urban/rural status was only available in the Komodo database.

Table S3: Distribution of between-dose intervals for the Komodo, TriNetX, and Cerner databases

n (%)	Komodo		TriNetX		Cerner	
	**BNT162b2**	**mRNA-1273**	**BNT162b2**	**mRNA-1273**	**BNT162b2**	**mRNA-1273**
Early (before recommended interval)	109,753 (0.8)	162,962 (1.2)	2,996 (0.3)	3,759 (2.3)	4,476 (0.4)	24,187 (3.6)
During recommended interval[a]	12,143,306 (88.6)	12,770,442 (91.6)	857,253 (89.3)	146,928 (91.4)	1,087,972 (90.7)	578,542 (86.6)
After the recommended interval, but within the allowable interval	1,369,058 (10.0)	849,551 (6.1)	95,988 (10.0)	8,620 (5.4)	102,674 (8.6)	59,483 (8.9)
Late (outside the allowable interval)	76,593 (0.6)	151,814 (1.2)	4,274 (0.4)	1,465 (0.9)	4,660 (0.4)	5,642 (0.8)

[a]Recommended interval is within ±4 days of the brand-specific recommended second dose schedule.

Table S4: Rates (%) of adherence and completion stratified by age (years) in the Komodo, TriNetX, and Cerner databases.

Age, years	18-22	23-26	27-39	40-64	65-74	75-84	>85	Unknown
**Adherence**								
**Komodo**								
Overall	75.7	76.9	78.4	80.1	81.8	82.6	81.5	43.2
BNT162b2	75.7	77.0	78.7	80.3	84.5	85.9	84.0	54.7
mRNA-1273	75.8	76.7	78.0	79.8	80.2	80.9	80.2	39.5
**TriNetX**								
Overall	81.2	80.4	81.3	86.1	88.3	88.0	87.1	83.7
BNT162b2	83.0	82.9	84.8	89.5	90.0	89.2	88.4	85.4
mRNA-1273	65.3	67.4	62.5	67.9	79.7	83.6	82.3	78.2
**Cerner**								
Overall	87.1	88.3	88.5	87.7	87.0	86.7	85.3	86.9
BNT162b2	89.5	90.6	90.8	90.6	91.1	90.7	88.7	88.6
mRNA-1273	79.4	82.1	82.7	82.2	82.2	82.2	81.7	83.6
**Completion**								
**Komodo**								
Overall	77.5	78.6	79.9	81.6	83.0	83.7	82.8	51.4
BNT162b2	77.0	78.3	79.8	81.5	85.3	86.6	84.8	58.7
mRNA-1273	78.2	78.9	80.0	81.7	81.7	82.3	81.7	49.1
**TriNetX**								
Overall	82.7	81.9	82.5	87.1	88.9	88.7	88.0	84.9
BNT162b2	84.4	84.1	85.8	90.1	90.5	89.7	89.1	86.1
mRNA-1273	67.4	70.7	65.7	70.9	81.5	85.1	84.2	81.0
**Cerner**								
Overall	88.0	89.3	89.5	88.9	89.7	90.1	88.5	88.1
BNT162b2	90.1	91.2	91.4	91.3	91.9	91.6	89.7	89.2
mRNA-1273	81.6	84.1	84.7	84.5	87.1	88.5	87.3	86.5

Table S5: Rates (%) of adherence and completion stratified by index month in the Komodo, TriNetX, and Cerner databases. Individuals included in Komodo and TriNetX databases were identified from December 2020 to June 2021. Individuals included in the Cerner database were identified from November 2020 to May 2021

Month	Nov '20	Dec '20	Jan '21	Feb '21	Mar '21	Apr '21	May '21	Jun '21
**Adherence**								
**Komodo**								
Overall	N/A	75.6	84.6	81.0	86.0	77.3	69.3	64.3
BNT162b2	N/A	73.1	82.4	84.4	87.9	78.3	70.5	64.0
mRNA-1273	N/A	78.7	85.7	79.0	84.3	76.1	67.1	65.3
**TriNetX**								
Overall	N/A	73.1	81.3	88.3	91.3	84.4	77.7	61.2
BNT162b2	N/A	77.2	85.1	90.3	92.8	86.1	80.3	66.2
mRNA-1273	N/A	63.3	73.5	79.8	78.2	60.6	52.0	19.6
**Cerner**								
Overall	95.1	93.2	89.1	87.8	88.5	83.9	74.5	N/A
BNT162b2	96.4	95.1	92.4	91.4	91.8	87.5	78.4	N/A
mRNA-1273	91.4	88.7	85.4	83.6	82.0	74.7	61.5	N/A
**Completion**								
**Komodo**								
Overall	N/A	77.2	86.4	82.4	87.2	78.8	71.1	65.8
BNT162b2	N/A	74.1	83.6	85.5	88.8	79.3	71.9	65.1
mRNA-1273	N/A	80.8	87.7	80.6	85.8	78.1	69.7	68.0
**TriNetX**								
Overall	N/A	73.9	83.1	88.8	91.8	85.9	78.5	61.8
BNT162b2	N/A	77.8	85.7	90.7	93.2	87.6	81.1	66.8
mRNA-1273	N/A	64.5	77.9	81.0	79.7	62.4	53.2	19.8
**Cerner**								
Overall	96.5	94.9	91.8	90.7	89.8	84.9	75.3	N/A
BNT162b2	97.2	95.9	93.4	92.1	92.4	88.0	78.8	N/A
mRNA-1273	94.1	92.5	89.9	88.9	84.7	76.9	63.8	N/A

Supplementary Figures

Figure S1: Recommended timeline for receipt of 2 doses of mRNA COVID-19 vaccines

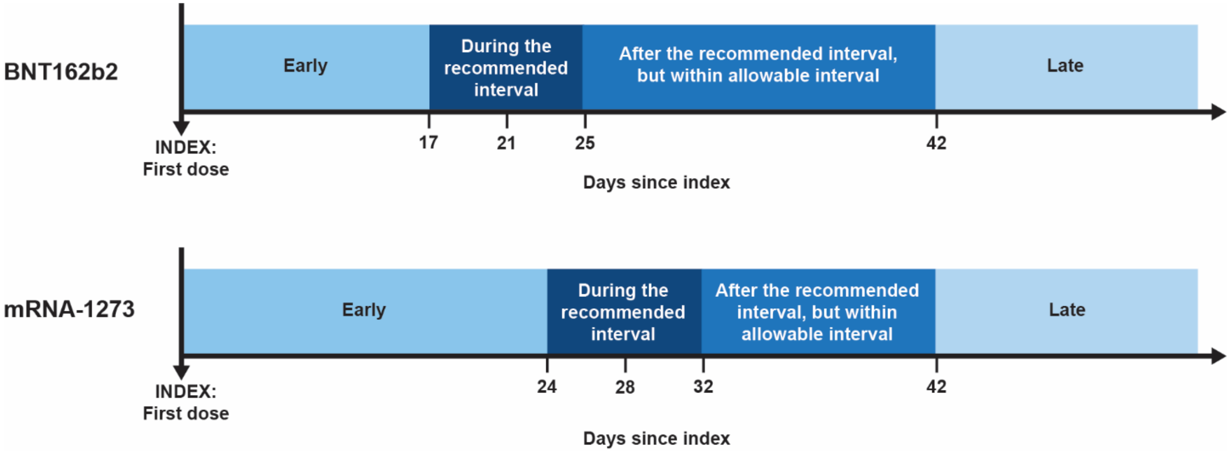

Figure S2: (A) Adherence and (B) completion stratified by urban/rural status (Komodo)

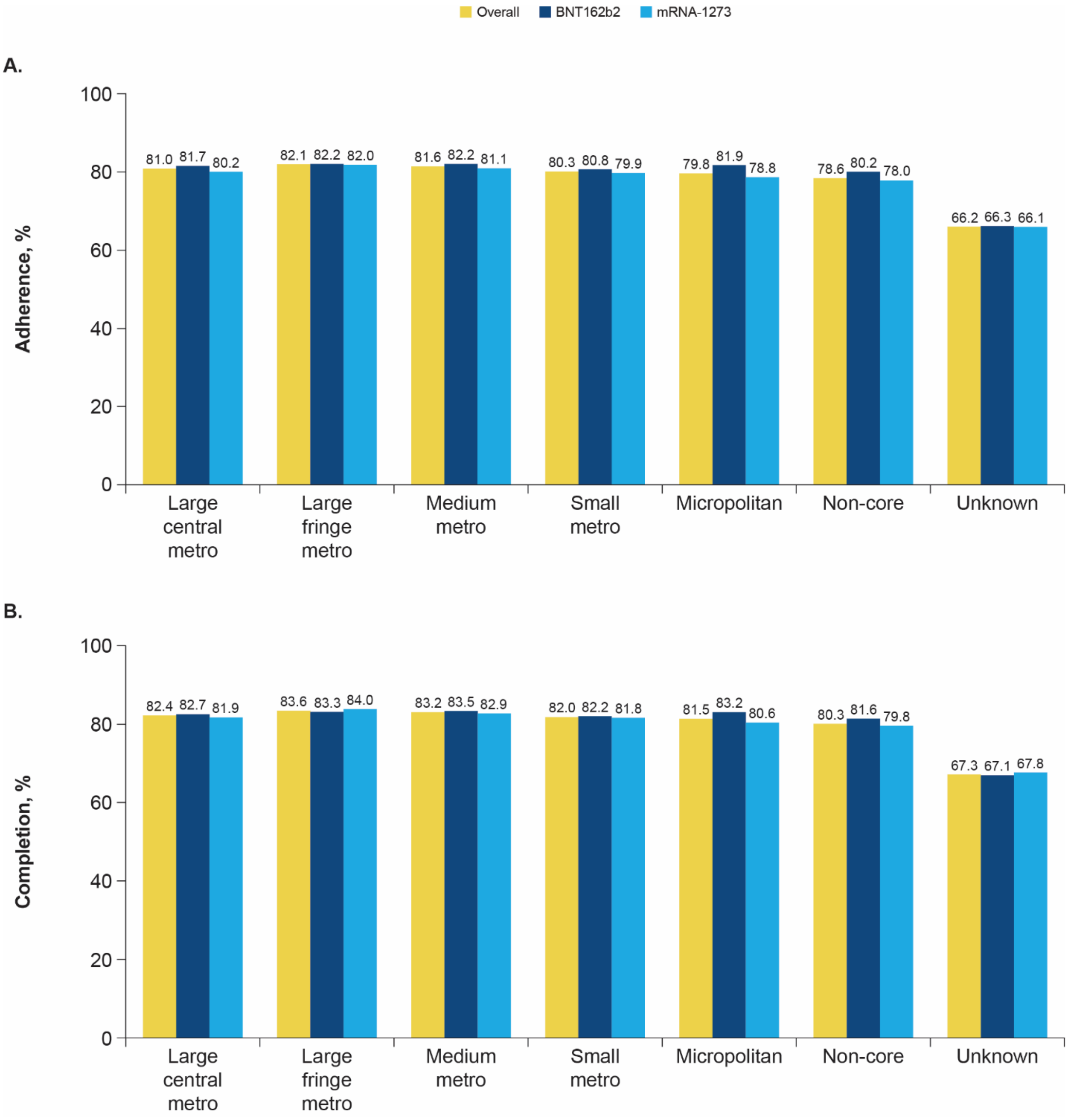

Figure S3: (A) Adherence and (B) completion by county in the Komodo database and (C) nationwide COVID-19 vaccination coverage by county showing the percent of the total United States population with at least one dose (Source: freely available from the Centers for Disease Control and Prevention website: https://covid.cdc.gov/covid-data-tracker/#county-view?list_select_state=all_states&list_select_county=all_counties&data-type=Vaccinations&metric=Administered_Dose1_Pop_Pct&null=Vaccinations).

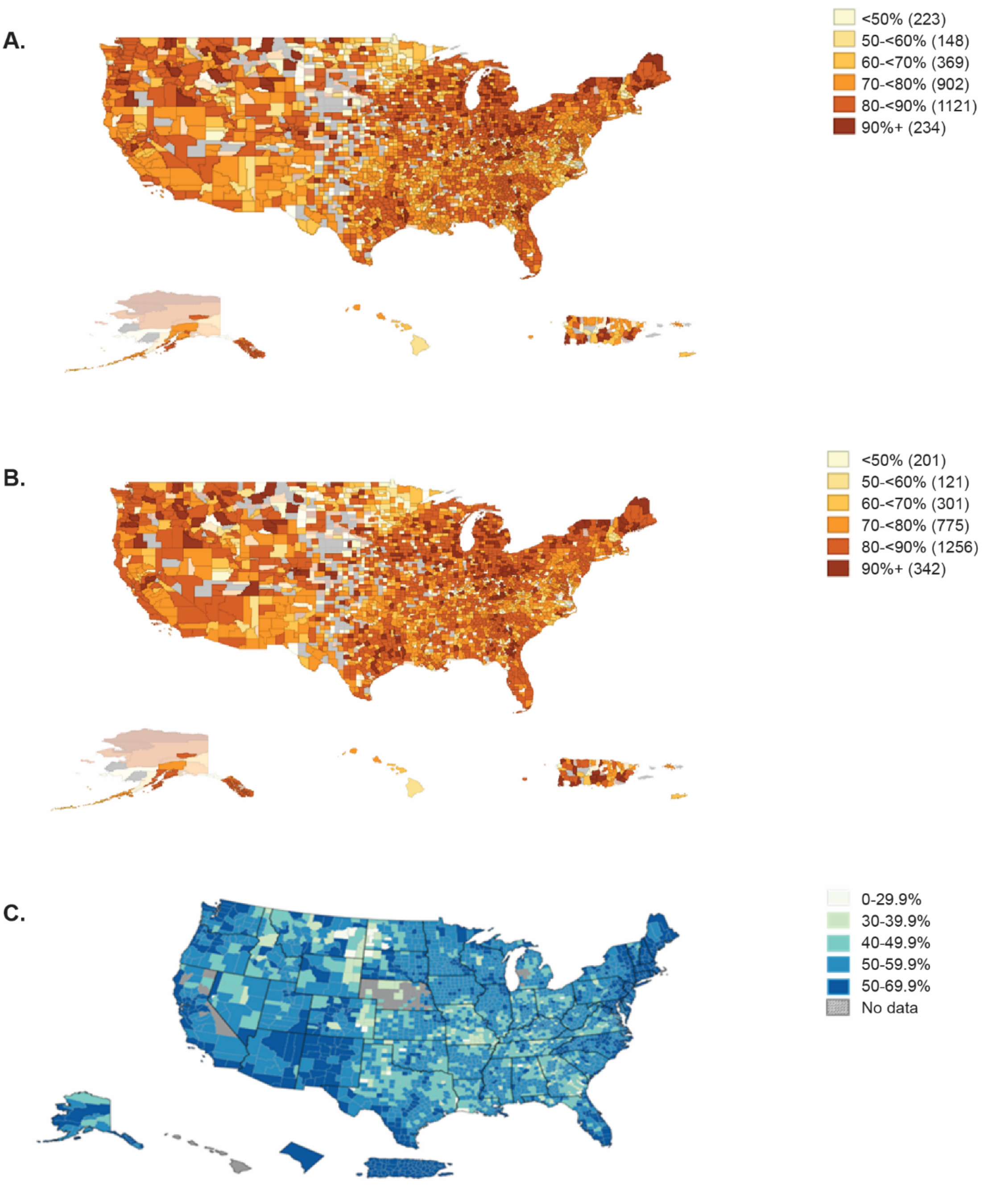

In panels (A) and (B), counties without data (n = 236) and counties with a patient count under 50 (n = 257) have been grayed out. Panel (C) is sourced from the Centers for Disease Control and Prevention; reference to specific commercial products, manufacturers, companies, or trademarks does not constitute its endorsement or recommendation by the U.S. Government, Department of Health and Human Services, or Centers for Disease Control and Prevention.

Figure S4: (A) Adherence and (B) completion by index month, stratified by age

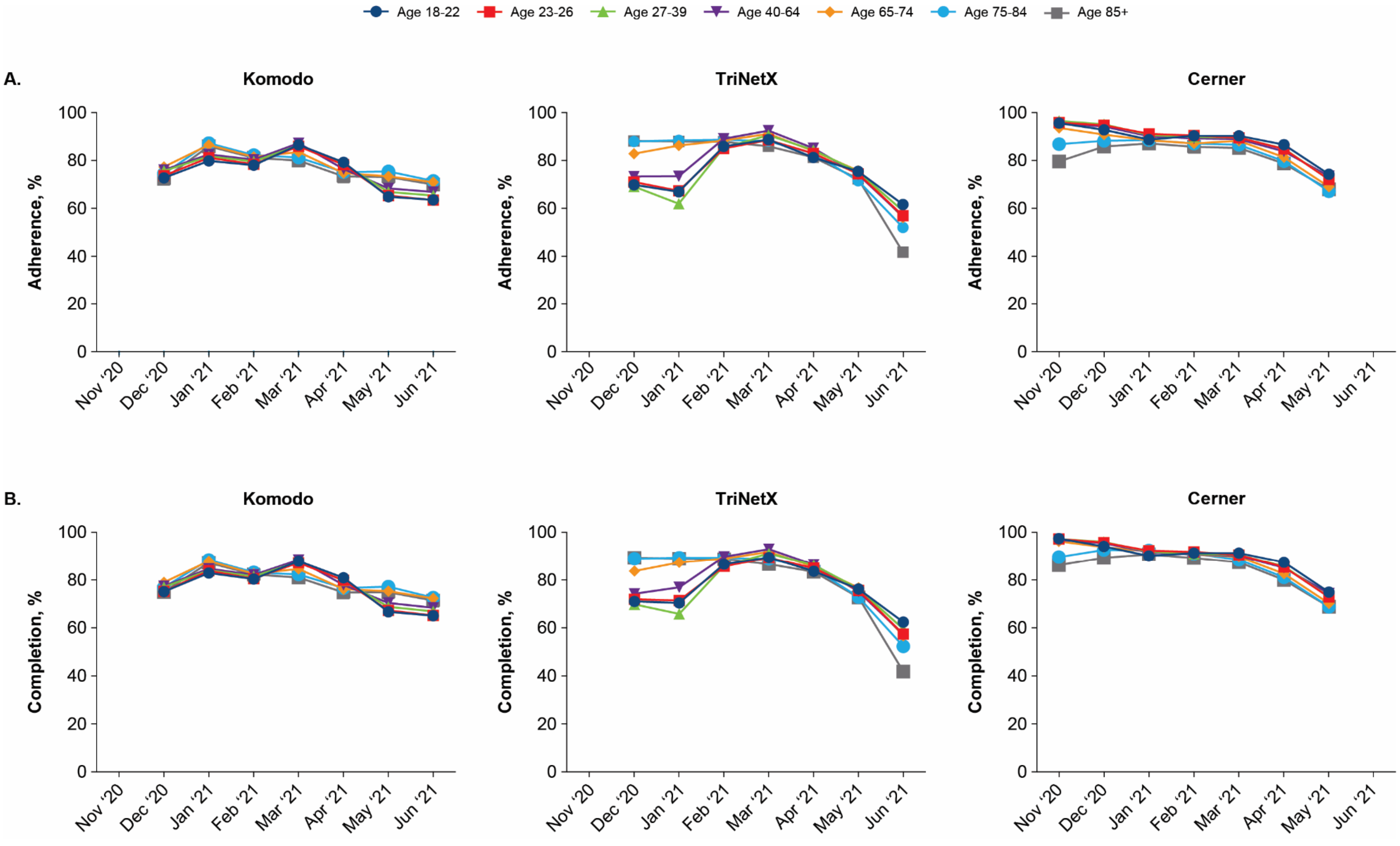

Figure S5: (A) Adherence and (B) completion by index month, stratified by race

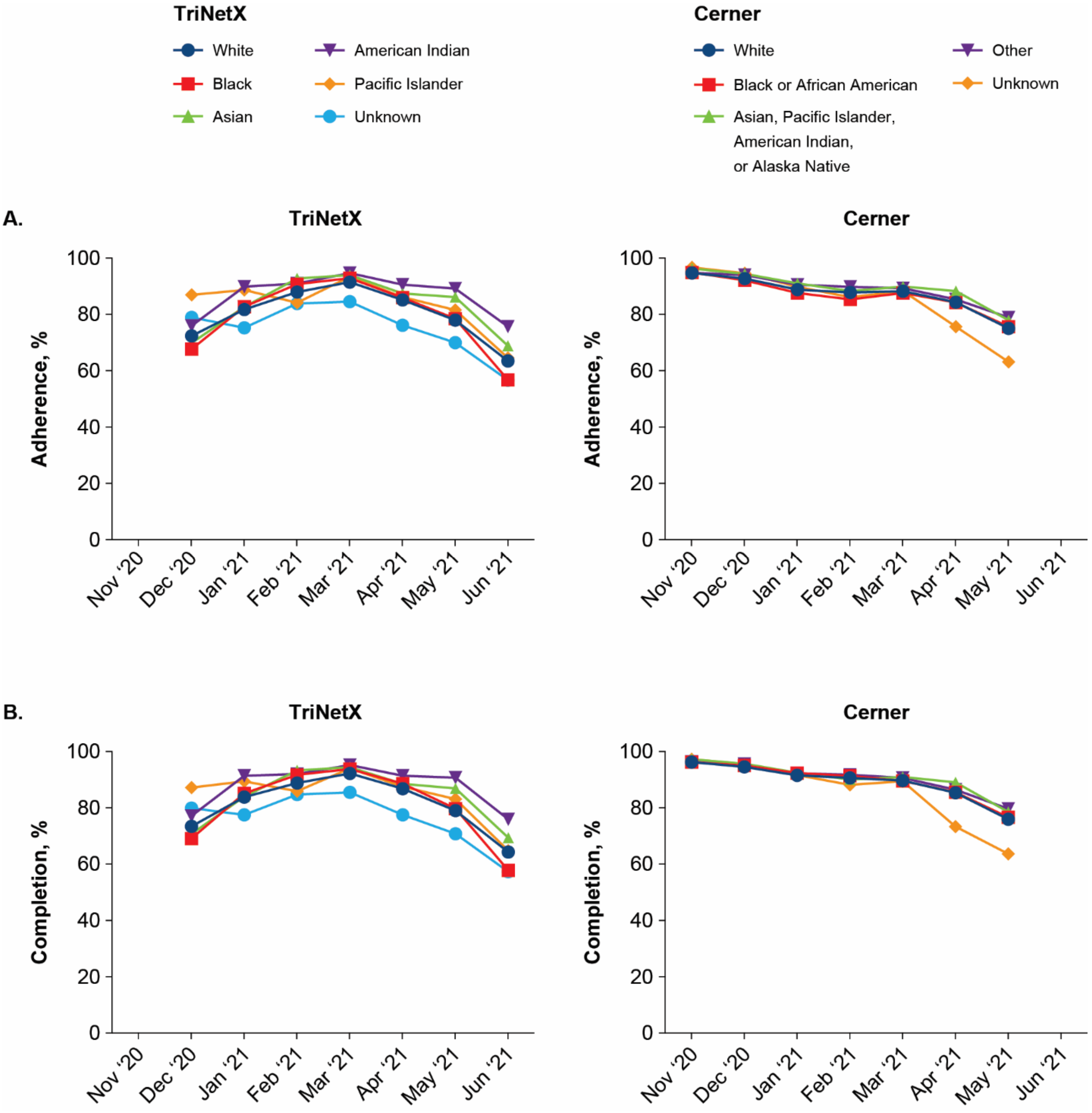

Supplement: Supplementary file 1 [file vaccines-10-01561-s001.zip › DeMartino_Revised Supplement_clean.pdf]
